# EGCG Inhibited Lipofuscin Formation Based on Intercepting Amyloidogenic β-Sheet-Rich Structure Conversion

**DOI:** 10.1371/journal.pone.0152064

**Published:** 2016-03-31

**Authors:** Shuxian Cai, Heng Yang, Kewu Zeng, Jing Zhang, Ni Zhong, Yingzi Wang, Jing Ye, Pengfei Tu, Zhonghua Liu

**Affiliations:** 1 State Key Laboratory of Natural and Biomimetic Drugs, School of Pharmaceutical Sciences, Peking University Health Science Center, Beijing, 100191, China; 2 Co-Innovation Center for Utilization of Botanical Functional Ingredients, Hunan Agricultural University, Changsha, 410128, China; University of Akron, UNITED STATES

## Abstract

**Background:**

Lipofuscin (LF) is formed during lipid peroxidation and sugar glycosylation by carbonyl-amino crosslinks with biomacrolecules, and accumulates slowly within postmitotic cells. The environmental pollution, modern dietary culture and lifestyle changes have been found to be the major sources of reactive carbonyl compounds *in vivo*. Irreversible carbonyl-amino crosslinks induced by carbonyl stress are essentially toxiferous for aging-related functional losses in modern society. Results show that (-)-epigallocatechin gallate (EGCG), the main polyphenol in green tea, can neutralize the carbonyl-amino cross-linking reaction and inhibit LF formation, but the underlying mechanism is unknown.

**Methods and Results:**

We explored the mechanism of the neutralization process from protein, cell, and animal levels using spectrofluorometry, infrared spectroscopy, conformation antibodies, and electron microscopy. LF demonstrated an amyloidogenic β-sheet-rich with antiparallel structure, which accelerated the carbonyl-amino crosslinks formation and disrupted proteolysis in both PC12 cells and D-galactose (D-gal)-induced brain aging mice models. Additionally, EGCG effectively inhibited the formation of the amyloidogenic β-sheet-rich structure of LF, and prevented its conversion into toxic and on-pathway aggregation intermediates, thereby cutting off the carbonyl-amino crosslinks.

**Conclusions:**

Our study indicated that the amyloidogenic β-sheet structure of LF may be the core driving force for carbonyl-amino crosslinks further formation, which mediates the formation of amyloid fibrils from native state of biomacrolecules. That EGCG exhibits anti-amyloidogenic β-sheet-rich structure properties to prevent the LF formation represents a novel strategy to impede the development of degenerative processes caused by ageing or stress-induced premature senescence in modern environments.

## Introduction

Lipofuscin (LF) is a brownish-yellow, electron-dense, and autofluorescent material that accumulates progressively with aging in postmitotic cells, such as in neurons, skeletal muscle cells, cardiac myocytes and retinal pigment epithelium. During carbonyl stress, the reactive carbonyl compounds of lipid peroxidation and sugar glycosylation induce misfolded carbonyl-amino crosslinks by binding with biomacrolecules and further form LF [1]. Over the past 50 years, lifestyle changes, new technologies and environmental pollution accelerate the reactive carbonyl compounds accumulation *in vivo*, which amplify the problem in public health today of age-related chronic diseases, most prominently obesity, cardiovascular, renal disorders, and neurodegenerative diseases.

The carbonyl-amino crosslinking products with conjugating and electron-deficient structures, have the highly active structures and can ceaselessly link and strangle with Cys, His, and Lys, and other nucleophilic amino acid residues of biomacrolecules. In the same way, the carbonyl-amino compounds bind with proteasome to disrupt cellular proteolytic systems and resistant to degradation, the process of which induce proteostasis deficiency, and might therefore be at the root of disease and aging-related functional losses [1–8].

Compared with oxidation products of proteins (ceroid), lipofuscin accumulates more slowly within postmitotic cells, and is more resistant to digestion. Using both free radical scavengers and antioxidants is inefficient on pre-formed reactive carbonyl compounds. Carbonyl scavengers have been developed to prevent carbonyl stress by inhibiting the formation of protein crosslinks, but most of the clinical trials prove to be unsatisfactory because of the side effects [9]. Moreover, previous studies show that the ability of inhibition of proteasomal activity is not significantly altered by chemically reducing carbonyls on the LF surface [10]. This characteristic indicates that carbonyl toxicity is not the only toxic factor of LF.

Our previous studies demonstrated that tea polyphenol (-)-epigallocatechin gallate (EGCG) and its methylated derivatives potently inhibited the formation of LF by neutralizing the carbonyl-amino cross-linking reaction [11–13]. However, the underlying molecular mechanism and whether these LF products on- or off-pathway aggregation products remain ambiguous. In this work, we demonstrated the deeply toxic mechanism of LF and the modulation of the LF formation pathway by EGCG in protein, cell and animal levels.

## Materials and Methods

### Ethical statement

The animals kept in a specific pathogen-free facility at Peking University Health Science Center. All the animals were handled accordance with the guidelines of national legislation for animal care. All experimental procedures and protocols were approved by the Institutional Animal Care and Use Committee of Peking University (No. SYXK (jing) 2011–0039). All surgery was performed under sodium pentobarbital anesthesia, and all efforts were made to minimize suffering.

### Materials

We purchased tea EGCG (≥98%) from Sigma. In addition to anti-GAPDH (Santa Cruz Biotechnology), the following primary antibodies were used for Western blot analysis: anti-multiubiquitin, anti-amyloid oligomer (A11) (Millipore), anti-amyloid fibrils (OC) (Millipore), and anti-sequestosome-1 (p62) (Epitomics). Western chemiluminescent horseradish peroxidase substrate was purchased from Millipore. Protease inhibitor mixture (Fermentas), BCA protein assay reagent (Piece) and radioimmunoprecipitation assay (RIPA) lysis buffer (Applygen Technologies Inc., Beijing, China) were also used in the experiments.

### Artificial lipofuscin (LF)

We prepared MDA-modified albumin of artificial LF with a typical LF-like fluorescence peak. Malondialdehyde (MDA) -modified human serum albumin (HSA) (Sigma) was prepared via a fresh MDA stock solution (10 mmol/L) prepared by hydrolyzing 1,1,3,3-tetramethoxypropane (Sigma) according to a method described by Kikugawa et al [14]. A solution containing HSA (1 mg/mL) and MDA (2 mmol/L) in PBS was incubated at 37°C in a sirocco-blasting drying trunk at different durations, and HSA (1 mg/mL) in PBS was prepared in the same way as the corresponding control. LF was subsequently filtered through a 0.22 μm-pore size membrane followed by filtration through Amicon Ultra 10K filter devices (Millipore) against water to remove any free form of MDA. Aliquots of LF were then measured and stored at −20°C.

### Fluorescence emission

Fluorescence measurements of the control or modified proteins were determined using FL Solutions 2.0 spectrofluorimeter [excitation wavelength (λex) = 395 nm and emission wavelength (λem) = 460 nm] to evaluate the effects of inhibiting the formation of LF-like fluorescence. The sample was diluted five-fold or 50-fold before the fluorescence data were analyzed. Alternatively, 10 μL aliquots of HSA or LF solution (1 mg/mL) were mixed with 90 μL of ThT (80 μg/mL) in TBS. The resulting fluorescence was measured immediately using a fluorescence plate reader (BioTek) at λex and λem of 440 and 485 nm, respectively.

### FTIR spectroscopy

Fourier transform infrared spectroscopy (FTIR) measurements were conducted according to our previous report [12]. Briefly, spectra ranging from 4000 cm^−1^ to 400 cm^−1^ were recorded at a reflectance mode with a spectral resolution of 4 cm^−1^, and each spectrum was accumulated 32 scans. To remove the absorbed water, samples were vacuum dried at −42°C for 24 h. OMNIC software (ThermoNicolet) was used to compose and analyze maps from the original, unprocessed spectra. The amide I band located at 1700 cm^−1^–1600 cm^−1^ was used to identify the protein secondary structure [15, 16]. Linear baseline was performed to the spectrum before the number and position of the component bands were estimated by applying Fourier self-deconvolution and secondary derivative calculations. From these analyses, the area of each component of the amide I band was quantified by a multiple Gaussian curve-fitting process in the region 1700 cm^−1^–1600 cm^−1^, and the area under the Gaussian curve quantified the relative percentage of the secondary structural elements. Correlations between amide I frequency and common protein structures: amide I maxima between 1675 cm^-1^–1695 cm^-1^ are generally assigned to antiparallel β-sheet/aggregated strands; and amide I maxima of 1660 cm^-1^–1670 cm^-1^, 1648 cm^-1^–1660 cm^-1^, 1640 cm^-1^–1648 cm^-1^, 1625 cm^-1^–1640 cm^-1^ and 1610 cm^-1^–1628 cm^-1^ are assigned to 3_10_-helix, α-helix, unordered strands, β-sheet and aggregated strands [15], respectively.

### Dot blot assay

To carry out dot blot assays to detect LF amyloid oligomers, 10 μL aliquots of LF (1 mg/mL) were applied to nitrocellulose membranes. The membranes were blocked for 1 h with 5% nonfat milk in TBST. The samples were then incubated with the anti-oligomer antibody A11 or anti-amyloid fibril antibody OC. After washing, a secondary antibody and the enhanced chemiluminescence substrate were used for detection [17].

### Cell assays

We obtained PC12 cells (rat pheochromocytoma) from the National Platform of Experimental Cell Resources for Sci-Tech (China). The PC12 cells were cultured in DMEM supplemented with 5% FBS, 10% horse serum, and 1% Penicillin-Streptomycinin in a 5% CO_2_-humidified environment at 37°C. Subsequently, 5×10^5^ cells were seeded into six-well plates to determine the misfolded aggregates pathway induced with LF in PC12 cells. The cells were either exposed or not exposed (control) to HSA, LF, or EGCG-degraded LF at a final concentration of 100 or 300 μg/mL for 48 h.

### D-galactose (D-gal)-induced brain-aged mouse model

We obtained KM mice, which were Healthy female, aged 7 weeks and weighed about 20 to 25 g, from the Department of Experimental Animals of Peking University Health Science Center, and we kept the animals in a specific pathogen-free facility at Peking University Health Science Center. After carrying out an acclimatization in the laboratory for a duration of one-week, we separated the mice into four groups randomly, and we used four cages to house the mice (8 animals per cage). The cages we used were equipped with standard rodent chow and of free accessible to water under temperature-controlled conditions (23 ± 2°C) at 70% humidity. We maintained animals in a reverse 12 h/12 h light/dark cycle (lights on at 7:00 AM and off at 7:00 PM). We subcutaneously injected the mice in the D-gal model groups and EGCG-treatment groups, respectively, with 3% D-gal at the dose of 150 mg/kg body weight once daily for 6 weeks, while we treated those in the control group with the same volume of normal saline. From the third week on, we gavaged EGCG at a dose of 10 or 20 mg/kg/d to the mice in the EGCG-treatment groups by the intragastric route, and we administered the same volume of vehicle (distilled water) to the mice in both the control and model groups for 4 weeks. We recorded the body weight of each mouse once a week. After finishing all treatments, we conducted euthanasia to the mice by intraperitoneal injection of 1% pentobarbital sodium (Merck) (30 mg/kg intraperitoneal injection) before we collected brain cortexes. We made all efforts to minimize the suffering of animals.

### Protein carbonyl contents

Protein carbonyl contents in mice cortices were measured with a carbonyl protein assay kit which was purchased from Nanjing Jiancheng Bioengineering Institute. The test was carried out strictly in accordance with the manufacturer’s instructions. The protein concentration was determined using a BCA protein assay reagent kit (Piece).

### Immunoblotting

We conducted Western blot analysis according to standard protocol. Cells were washed, harvested, and lysed in sequence; washing was performed twice with ice-cold 1×PBS, and lysing in RIPA buffer with protease inhibitor mixture. We lysed the mixture in an ice bath for 30 min and then centrifuged for 15 min at 15,000×g at 4°C, and discarded the insoluble debris. A BCA Protein Assay Reagent was used to determine the protein concentration. Cell lysate (20–40 μg) was subjected to SDS-polyacrylamide gel electrophoresis with a concentration of 8%–15%, and transferred to nitrocellulose membranes (Millipore) [18], which were blocked with non-fat milk (of 5%) and incubated with primary antibodies for 24 h at 4°C. The membranes were then incubated with secondary antibody (Epitomics) of HRP-conjugation for 1 h at room temperature after three washes in TBST. A Kodak Digital Imaging System was used to scan the proteins on a chemiluminescent substrate to visualize the proteins.

Mice cortices placed individually in a 2 mL glass homogenizer with RIPA lysis buffer (Applygen Technologies Inc., Beijing, China) containing a protease inhibitor mixture (Fermentas). The mixture was lysed in an ice bath for 30 min and centrifuged for 15 min at 15,000 ×g at 4°C. The protein concentration was determined using a BCA protein assay reagent kit (Piece). Western blot analysis was performed as described above.

### Transmission electron microscopy (TEM) analysis

A JEM1400 transmission electron microscope was used to examine and photograph the aliquots of protein samples (0.5 mg/mL), which were absorbed to carbon-coated copper grids for 1 h and negatively stained with 2% uranyl acetate for 1 min [19].

Before TEM measurements, a mixture of 2.5% glutaraldehyde and 2% paraformaldehyde in 0.1 M PBS (pH 7.4) was used to fix the cerebral cortexes of mice. Afterwards, the cerebral cortex in the identical portion of each mouse was obtained and fixed in 1% osmium tetroxide for 1 h. Ultrathin sections were cut from these samples after subjected to treatments in sequence, namely, dehydration, embedment in epoxy resin and trim. Uncoated mesh copper grids were used to mount sections with a thickness of 40 nm—60 nm, which were constructed with uranyl acetate for 30 min and lead citrate for 2 min. JEM1400 transmission electron microscope was utilized to examine and photograph the section samples.

### Statistical analysis

The GraphPad Prism version 6.0 (San Diego, CA, USA) was used to analyze data and create graphs. All experiments were repeated three times. All data were expressed as mean ± standard deviation (SD) of the mean from three independent experiments. Student’s *t*-test or analysis of variance (ANOVA) was used to determine the statistical differences. The value of *P* < 0.05 was considered statistically significant.

## Results

### EGCG prevented β-sheet-rich amyloidogenesis of lipofuscin (LF)

**Fig 1:**
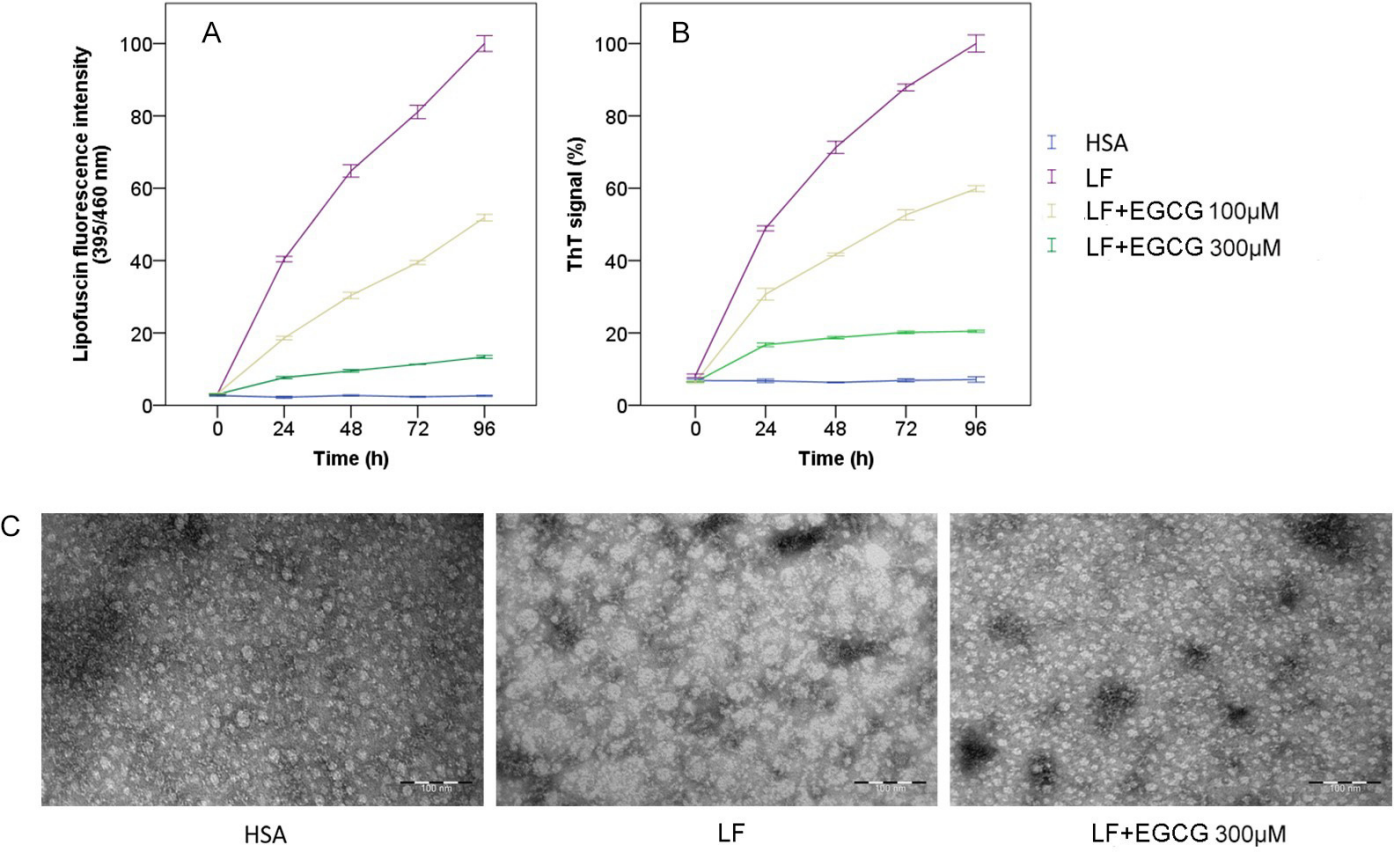
(-)-Epigallocatechin gallate (EGCG) prevented β-sheet-rich amyloidogenesis of lipofuscin (LF). (A) Effect of EGCG on LF formation as measured by emission of LF-like fluorescence intensity at 460 nm in MDA-modified HSA artificial LF (1 mg/mL) reaction system. (B) Effect of EGCG on LF β-sheet-rich structure formation by measuring Thioflavin T (ThT) fluorescence emission at 485 nm. (C) Analysis of sample aggregation reactions by transmission electron microscopy (TEM) in different groups after 96 h of incubation. Scale bars represent 100 nm. Data are expressed as means ± SD, n = 3.

First, we evaluated the effect of EGCG on LF formation using spectrofluorometry. MDA-modified HSA artificial LF (1 mg/mL) reaction system was incubated in the presence and absence of EGCG, and we monitored carbonyl-amino crosslinks by measuring LF-like fluorescence (fluorescent 1-amino-3-iminopropene crosslinks) emission at 460 nm [20]. The results showed that EGCG remarkably inhibited LF-like fluorescence intensity (Fig 1A), which indicated that EGCG could neutralize the carbonyl-amino crosslinking reaction in the MDA-modified HSA artificial LF reaction system.

We then monitored LF β-sheet-rich amyloid structure by measuring Thioflavin T (ThT) fluorescence emission at 485 nm. In the absence of EGCG, we observed ThT-positive aggregates after a lag phase of 96 h, indicating the presence of β-sheet-rich structure. By contrast, the ThT-positive aggregates were suppressed in the presence of EGCG (300 μM relative to the LF reaction system), with a significant reduction in fluorescence signal (~10%) (Fig 1B).

In another experiment, we investigated the effect of EGCG on LF amyloidogenesis by negative-stain TEM. In the LF group, we observed the formation of predominantly fibrillar structures with a diameter of ~50 nm nucleation polymer or linear structure, which was consistent with the result of the ThT assays. By contrast, EGCG markedly reduced fibril assembly, promoted spherical oligomers with an average diameter of ~20 nm, which was similar to that in the HSA group (Fig 1C). Thus, EGCG [300 μM relative to LF (1 mg/mL) reaction system] efficiently prevented the amyloidogenesis of LF and maintained the original spherical oligomers form of HSA.

### FTIR analysis of lipofuscin (LF) β-sheet-rich amyloidogenesis structure

**Fig 2:**
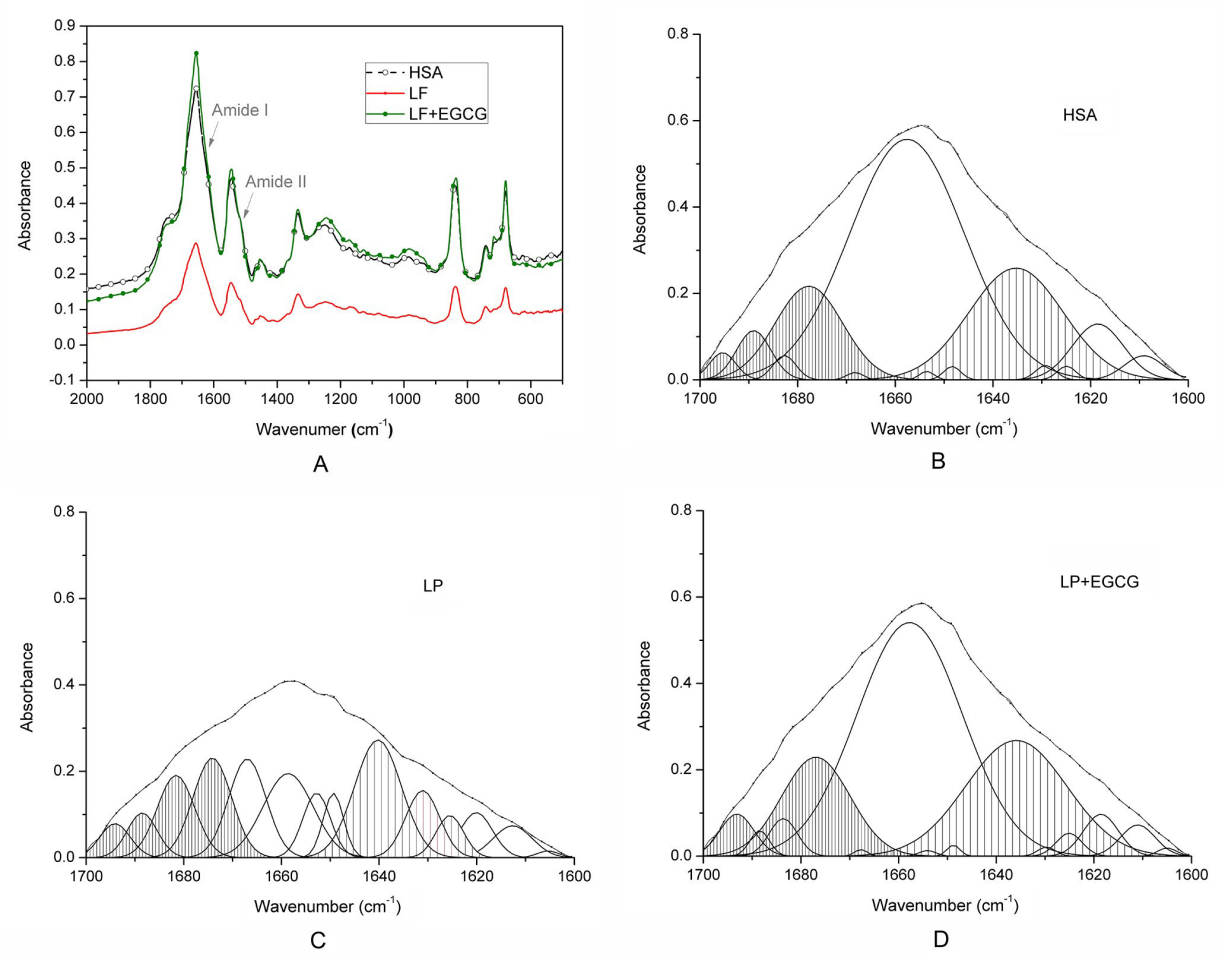
FTIR analysis of lipofuscin (LF) β-sheet-rich amyloidogenesis structure. (A) The FTIR spectra of the protein products in different treatment groups. (B-D) The peaks from a Gaussian curve fitting of the FTIR spectra of the amide I band (normalization processing) of protein products in different treatment groups. Sparse shading is β-sheet structure (1625 to 1640 cm^-1^), and dense shading is antiparallel β-sheet/aggregated strands structure (1675 to 1695 cm^-1^). LF and LF+EGCG represent LF and EGCG (300 μM) treated LF groups after 96 h of incubation, respectively.

Previous studies have indicated that β-sheet formation is a crucial early step in amyloidogenesis [20]. We speculated that EGCG blocks LF formation by preventing amyloidogenic β-sheet structure formation. To examine this hypothesis, we performed FTIR experiments with EGCG-treated and untreated groups. Infrared spectroscopy can be used to probe changes in secondary structure of the protein backbone [21]. The results showed that the absorbance of the amide I band sharply decreased after HSA was incubated with MDA. After EGCG treatment, the absorption spectrum of the treatment group was almost consistent with that of the HSA control group (Fig 2A). The amide I absorption contains contributions from the C = O stretching vibration of the amide group (about 80%). The stronger the hydrogen bond involving the amide C = O, the lower the electron density in the C = O group and the lower the amide I absorption appears [15]. The FTIR results indicated that EGCG could change the secondary structure of the LF backbone.

To understand the changes in protein structure, we quantitatively analyzed the secondary structure of the amide I band [22–24]. We identified peak centers followed by Gaussian fitting to quantify the deconvolved peaks in the FTIR spectra. As seen in Fig 2C and Table 1, the major component of the FTIR spectrum was centered at around 1640 cm^−1^, and the area integral percentage of LF β-sheet was 24% (sparse shading). The area integral percentage of antiparallel β-sheet/aggregated strands structure (dense shading) of LF was 25.113%, which was the major component of the second structure of LF. The area integral percentage of the protein second structures of EGCG treatment groups were similar to those of the HSA control groups, the FTIR spectrum was centered at around 1658 cm^−1^, and the major component of the second structure was α-helix (Fig 2B and 2D, Table 1). Our FTIR results showed that LF had substantial β-sheet-rich amyloidogenesis structure, and that EGCG inhibited LF amyloidogenic β-sheet-rich structure conversion. This was consistent with the results of ThT fluorescence assays.

**Table 1:** (-)-Epigallocatechin gallate (EGCG) inhibited the formation of lipofuscin (LF) β-rich amyloidogenesis structure. LF and LF+EGCG represent LF and EGCG (300 μM) treated LF groups after 96 h of incubation, respectively.

	Aggregated strands	α-helix	3_10_-helix	β-sheet	Unordered	Antiparallel β-sheet/aggregated strands
HSA	7.732	53.195	0.236	20.682	0.436	19.534
LF	10.467	21.089	9.983	24.232	4.025	25.113
LF+EGCG	6.297	49.957	0.173	23.736	0.291	17.719

Note: secondary structure of LF was predicted from FTIR.

### EGCG stimulated assembly of off-pathway oligomers

**Fig 3:**
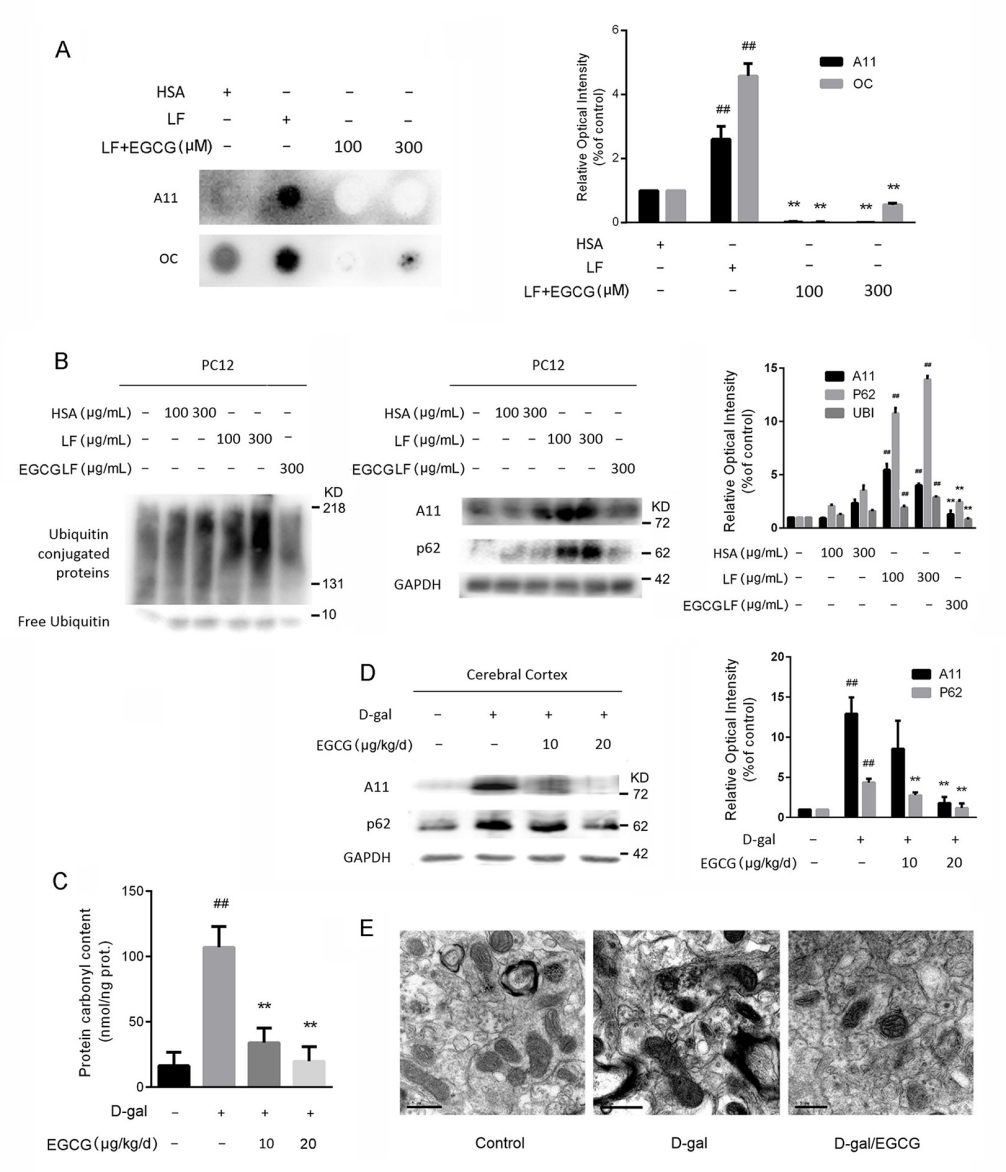
(-)-Epigallocatechin gallate (EGCG) stimulated assembly of nontoxic, off-pathway oligomers. (A) Detection of amyloid oligomers by A11 antibody and fiber-specific OC antibody in HSA, lipofuscin (LF) or EGCG LF oligomers. EGCG LF oligomers were produced with 100 or 300 μM concentrations of EGCG for 96 h at 37°C. (B) Western blot detection of ubiquitin-conjugated proteins (UBI), A11, and p62 expression of PC12 cell which incubated with HSA, LF or EGCG LF oligomers (100 or 300 μg/mL) for 48 h. (C) The inhibitory effect of EGCG (10 or 20 μg/kg/d) on the formation of carbonylated proteins in the brain aging cortex. (D) Western blot detection of A11 and p62 expression from the brain cortex lysate of different treatments. (E) Comparison of LF and mitochondria of cortical neurons in different treatment groups. ^##^*P*<0.01 represents the statistical significance vs. control group; **P<0.01 represents statistical significance vs. D-gal-treated group, n = 3.

We further tested whether amyloidogenic oligomers were generated in EGCG-treated or untreated aggregation reactions with conformation-specific antibody A11 and amyloid fibrils with antibody OC. We performed dot blot assays and found evident amyloidogenic oligomers and fibril assembly in LF samples. Moreover, the formation of A11- and OC-reactive amyloid oligomers was efficiently suppressed by EGCG (100 or 300 μM) addition after an incubation period of 96 h at 37°C but not in the absence of EGCG (Fig 3A).

We also carried out assays to investigate whether EGCG-generated oligomers were toxic for mammalian cells. We obtained HSA, β-sheet-rich amyloid LF and EGCG-incubated products in vitro. Then we added these structures with different concentrations to PC12 cells. We used ubiquitin-conjugated proteins (UBI) and p62 proteins as diagnostic tools as reported [25–27] to characterize the aggregates formation in cells. Results of Western blot analysis showed a sharp increase in the levels of A11, UBI, and p62 from the cell lysate following LF treatment for 48 h. By contrast, EGCG-incubated products remarkably decreased the level of ubiquitin-aggregated proteins (Fig 3B). This phenomenon indicated that the EGCG-stabilized structure was less noxious than β-sheet-rich LF oligomers in cell-based assays.

Finally, we examined the effects of EGCG on the formation of LF aggregates in animal brain-ageing model. D-galactose (D-gal)-treated mice have been used as a mature model for studying the mechanism of oxidative stress and inflammatory-induced brain aging, neurodegenerative changes, and screening neuroprotective drugs [28–30]. Protein carbonyl contents of the cerebral cortices were quantitatively determined and compared. Protein carbonyl content of D-gal-induced brain aging cortex was 107.1 nmol/ng prot, which was 5.7 times higher than that of the negative control group. EGCG-treated groups exhibited high inhibitory activity (Fig 3C). Results of Western blot analysis showed that A11 and p62 levels in the brain cortex of the D-gal-treated group increased, and EGCG decreased the relevant protein levels of the aggregates (Fig 3D). Results of TEM showed that the sizes of LF particles in the cortical neurons increased, and mitochondria were sparsely distributed and exhibited vacuolar degeneration with disrupted cristae in the D-gal-treated group. Degenerative changes were alleviated after EGCG treatment (Fig 3E).

## Discussion

Our data revealed that LF aggregates had amyloid-β amyloidogenesis structures, such as A11- and OC-reactive amyloid oligomers and antiparallel β-sheet/aggregated strands structure. Moreover, these compounds excited the misfolding aggregation pathway in both cell and animal levels. Small-molecule EGCG triggered β-sheets to redirect LF formation to off-pathway species.

Our results from ThT fluoroscopy and FTIR showed that the amount of β-sheet-rich amyloid structure increased sharply during LF formation (Figs 1B and 2C, Table 1). Dot blot and TEM data indicated that the carbonyl-amino crosslinks promote protein conversion into β-sheet-rich A11- and OC-reactive oligomers (Figs 1A, 1C and 3A), independent of the primary amino acid sequence. Western blot analysis further confirmed our presumption that LF excited the protein misfolding pathway in cells by increasing toxic β-sheet amyloid structures, UBI and p62 modified proteins, which further induced aggregates formation in cells (Fig 3B). Our studies confirmed the opinion that carbonyl stress induced carbonyl-amino crosslinks attribute the aberrant posttranslational modifications, altering the long-term function of autophagy (Fig 3C–3E), in this way to make proteolysis deficiency [31].

Our FTIR data also confirmed that carbonyl-amino crosslinking may enhance antiparallel β-sheet/aggregated strands structure formation (Table 1). The amide I absorbance is from C = O stretching vibrations of the polypeptide backbone carbonyls and is an established indicator of secondary structure. The dihedral angles of a polypeptide chain determine the chain geometry, which dictates the length and direction of hydrogen bands involving amide C = O and N-H groups. Variations in the length and direction of hydrogen bonds result in variations in the strength of the hydrogen bond for different secondary structures, which in turn produce characteristic electron densities in the amide C = O groups, resulting in characteristic amide I frequencies. The stronger the hydrogen bond involving the amide C = O, the lower the electron density in the C = O group and the lower the amide I absorption appears. Our FTIR results showed that the absorbance of amide I band sharply decreased in the LF group (Fig 2A), which indicated that the lowest amide I frequency to occur in extended polypeptide chains connected by hydrogen bands involving amide C = O and N-H groups. Fibrils with antiparallel β-sheet structures exert distinct pathogenic effects [23, 32–34]. This phenomenon is probably the reason why the prevention of aldehyde formation on the LF surface does not significantly alter the ability of LF to inhibit proteasomal activity [10].

**Fig 4:**
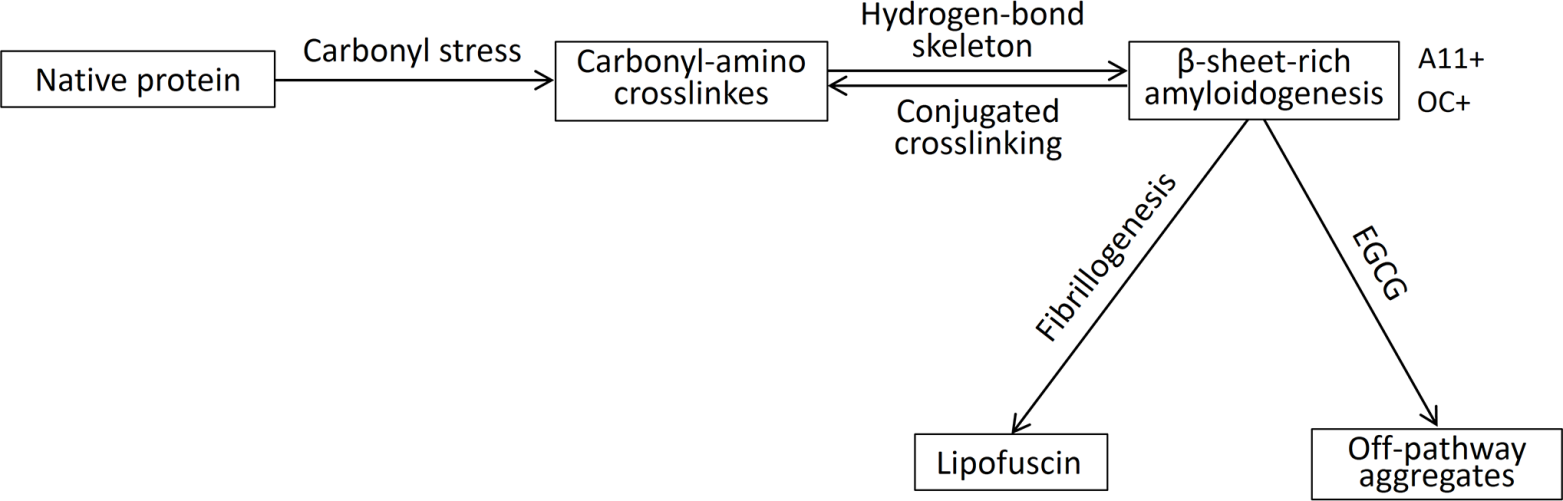
Working models of effects of (-)-Epigallocatechin gallate (EGCG) on lipofuscin (LF) amyloidogenesis. Native proteins tend to aggregate to form β-sheet-rich amyloidogenesis structures (A11-positive and OC-positive) by carbonyl-amino crosslinking under carbonyl stress. The carbonyl-amino crosslinks and misfolded β-sheet-rich amyloid structures activated each other, a pathological feedback loop resulting in LF formation and accumulation which continuously supplied by native protein as raw material. EGCG prevented β-sheet-rich amyloidogenesis formation by inducing off-pathway oligomers protein modifications, in this way to inhibit LF formation.

The above results indicated that when carbonyl-amino crosslinks and misfolded β-sheet-rich amyloid structures activated each other, a pathological feedback loop involving LF formation and accumulation. The carbonyl-amino crosslinking products with conjugating and electron-deficient structures lead to the formation of rigid double plane structure formation. The amide group is generally considered to be planar (ω = 180), due to resonance and tautomerism, and results in the C-N bond assuming partial double-bond character due to electron flow from the C = O group, which is conducive to the further formation of the intermolecular hydrogen bands involving amide C = O and N-H groups on the nucleophilic amino acid residues of biomacrolecules and so as to promote β-sheet structure formation and stabilize the extended aggregates. A previous study show that, the N-H and C = O atoms, in antiparallel β-sheet, must belong to the same residue of the upper and the lower chain interaction for a pair of neighbouring h-bonds to be counted as one h-bond pair [22]. The β-sheet-rich amyloid structures, especially antiparallel β-sheet, creates the conditions for the further formation of carbonyl-amino compounds (Fig 4). Amyloidogenic β-sheet-rich structures originated from the carbonyl-amino crosslinks was a key inducement to the direct and rapid conversion of native state proteins to soluble oligomers to insoluble amyloid. The conversion of soluble oligomers of most common proteins into amyloidogenic β-sheet-rich LF agreed with the current consensus that every protein probably has native and fibrous states, and most human proteins can mediate the formation of amyloid fibrils in despite of the implication of specific proteins in certain diseases [19, 35–37].

Our results demonstrated that EGCG can inhibit the formation of LF by intercepting amyloidogenic β-sheet-rich structure conversion. Our in vitro studies indicated that EGCG redirected LF formation by preventing the formation of toxic, β-sheet-rich amyloid or fibrillar oligomers (Fig 1, Fig 2 and Table 1). Our in vivo studies indicated that EGCG prevented LF toxicity by stimulating assembly off-pathway oligomers protein modifications, aggregation, and toxicity, and protected proteolytic systems (Fig 3). On the basis of the experimental data presented here, we concluded that EGCG prevented amyloidogenic β-sheet-rich and antiparallel structure conversion into LF (Fig 4). These results were in agreement with the previous finding that EGCG can redirect misfolded protein intermediates to off-pathway species, such as α-synuclein, amyloid-β, and huntingtin [17, 38]. EGCG is an aromatic compound with many hydroxyl groups, which may further stabilize protein backbone by hydrogen bonds, but not covalent binding [17, 39]. Moreover, the gallate groups, especially the galloyl groups (at 3-C position) of EGCG, are essential for physiologic activity by increasing the targets of misfolded hydrophobic parts of biomacromolecules, but not native ones [11–13].

In summary, our data showed that polyphenol EGCG obviously alleviated the LF formation on the protein, cell, and animal levels. EGCG exhibiting anti-amyloidogenic β-sheet-rich LF property maybe represent a novel strategy to impede the progression of age-related macular degeneration. Long-term drinking of tea may be one of the effective strategies for the deceleration of the progression of degenerative disorders and extend healthy life spans in modern environments.

## Abbreviations

LF: Lipofuscin
EGCG: (-)-epigallocatechinegallate
HSA: Human Serum Albumin
FTIR: Fourier Transform Infrared Spectroscopy
ThT: Thioflavin T
TEM: Transmission Electron Microscope
UBI: Ubiquitin-conjugated Proteins
D-gal: D-galactose
